# Dialogue intervention for youth amidst intractable conflict attenuates neural prejudice response and promotes adults’ peacemaking

**DOI:** 10.1093/pnasnexus/pgac236

**Published:** 2022-10-14

**Authors:** Jonathan Levy, Moran Influs, Shafiq Masalha, Abraham Goldstein, Ruth Feldman

**Affiliations:** Ivcher School of Psychology, Reichman University, 8 Ha'universita Street, Herzliya 4610101, Israel; Department of Neuroscience and Biomedical Engineering, Aalto University, 02150 Espoo, Finland; Ivcher School of Psychology, Reichman University, 8 Ha'universita Street, Herzliya 4610101, Israel; Ono Academic College, Kiryat Ono 55000, Israel; Gonda Multidisciplinary Brain Research Center and Department of Psychology, Bar-Ilan University, Ramat-Gan 5290002, Israel; Ivcher School of Psychology, Reichman University, 8 Ha'universita Street, Herzliya 4610101, Israel; Child Study Center, Yale University, New Haven, CT 06510, USA

**Keywords:** intergroup conflict reduction, Implicit Association Test (IAT), Magnetoencephalography (MEG), alpha oscillations, prejudice

## Abstract

Humans’ dependence on group living has led to the formation of tenacious, often nonconscious negative perceptions of other social groups, a phenomenon termed “intergroup bias” that sustains one of the world’s most imminent problem: intergroup conflicts. Adolescents’ participation in intergroup conflicts has been continuously on the rise, rendering the need to devise interventions that can mitigate some of their deleterious effects on youth an urgent societal priority. Framed within the Israeli–Palestinian conflict and targeting youth, we implemented a dialogue-enhancing intervention for adolescents (16 to 18 years) reared amidst intractable conflict that builds on social synchrony and the neurobiology of affiliation. Implementing a randomized controlled trial design, before and after the 8-week intervention adolescents underwent magnetoencephalography to assess a neural marker of implicit prejudice and interviewed on their attitudes toward the conflict. Adolescents who received the intervention showed attenuation of the neural prejudice response, as indexed by sustained occipital alpha that was significantly reduced at post-intervention and adopted attitudes of peacemaking. Change in the neural prejudice response predicted attitudes of compromise and support in peacebuilding 7 years later, when young adults can already engage in active civil duties and responsibilities. These results underscore adolescence as a window of opportunity for enhancing inter-group dialogue and demonstrate the long-term associations between the neural evaluation of prejudice and self-reported measures of proclivity for compromise and peace in the context of an intractable century-long conflict.

Significance Statement One of civilization’s most important challenges is finding evidence-based ways to minimize intergroup conflicts. Framed within the Israeli–Palestinian conflict and targeting youth, the current study implemented dialogue-enhancing intervention based on the neurobiology of affiliation. Magnetoencephalography and interviews were used to assess a neural marker of prejudice and attitudes toward the conflict before and after intervention. Following intervention youth showed attenuation of the neural prejudice response and increased support in peacebuilding. Critically, change in the neural prejudice response predicted adults’ attitudes toward compromise and peacemaking 7 years later. Results, demonstrating very long-term prediction of intergroup intervention effect, underscore the utility of neuroimaging and frame adolescence as window of opportunity for dialogue and its associated neurobiology in the context of intractable conflicts.

## Introduction

A central features of humans’ evolutionary-based reliance on group living is the negative perceptions they hold of other social groups, a phenomenon termed “intergroup bias” (or, alternatively, “ingroup bias”), which underpins one of the world’s most imminent problems: intergroup conflict (1). The Israeli–Palestinian conflict is an intractable intergroup conflict that has led to over a century of immense suffering, deep hatred, marked prejudice, and inability to make actual steps toward compromise and peace (2). From a young age, children of both groups display negative attitudes and hatred toward the outgroup, exhibit little empathic behavior, show intense prejudice (3), and attenuate their neural empathic response to the pain of outgroup (4, 5) [see ref. (6) for such attenuation in adults]. These, in turn, trigger intense fears that limit the opportunities for dialogue among the two groups, despite the fact that dialogue is the only way to move out of this long-lasting deadlock (7). Overall, a wide range of interventions have been developed to reduce intergroup conflicts over the past 60 years (8) and a variety of interventions have similarly been implemented between Jews and Arabs in Israel (9). Still, despite the massive effort to reduce hatred and enhance peace (10), results have been mixed; some interventions reported negative effects (11), others yielded positive findings (12), and still others showed mixed outcomes (13). In the current study, we utilized the Tools of Dialogue© intervention (14–16), a validated intervention for youth reared amidst intractable conflicts guided by the *biobehavioral synchrony* conceptual frame. *Biobehavioral synchrony* is a bottom-up, behavior-based mechanism that describes how the coordination of nonverbal social signals between two humans, including shared gaze, joint affect, or synchronized movement, lead to the coordination of physiological processes between social partners and tune the social brain for greater empathy, sharing, and mentalization. The model also describe how *biobehavioral synchrony* matures from its first expression within the mother–infant bond to mechanisms that sustain group living and allow greater collaboration, empathy, and sharing within social groups and how the neurobiological basis of hatred and derogation can be modified through synchronized social action.

To date, studies assessing outcomes of intergroup interventions relied solely on self-report and behavioral measures (17), and none, to our knowledge, utilized neural measures to pinpoint changes in brain response following intervention, particularly long-term effects. Since self-reports are subjective and behavioral indices, such as response time (RT), are too broad to identify specific processes, authors have advocated the use of neuroimaging to specify mechanisms of change in the context of intergroup conflict (18). Cumulative evidence indicates that neuroimaging can uniquely assess cognitive and affective processes impacted by the intervention and provide better prediction of intervention outcome as compared to traditional measures (19), highlighting their promise as venue for future research (20). Three recent studies provide initial support to this approach. Hein and colleagues studied under controlled lab conditions the effects of negative reinforcement learning on intergroup empathy in the Swiss immigrant context and found that during the experimental session, a positive learning experience towards outgroup members resulted in enhanced neural empathic response towards representations of outgroup. Farmer and colleagues found that positive intergroup contact did not link with implicit bias on the Implicit Association Test (IAT), but was reflected by reduced neural intergroup bias; however, these results were correlational (21). Finally, Valencia and colleagues utilized an intervention training in the context of ex-combatants reintegration and found that the intervention triggered covert neurofunctional reorganization even when in the absence of overt behavioral improvements (22).

Prejudice reflects the nonconscious, often irrational preference for one’s social group over other groups (8). Social neuroscience has made considerable contribution to our understanding of prejudice (23), particularly the mechanisms and processes implicated in the perception and expression of prejudice and the regulation of intergroup emotions and behaviors (24). One subtle type of prejudice is the implicit intergroup bias, a nonconscious bias that persists even when the individual explicitly denies it (25). The most widely used measure to evaluate implicit intergroup bias is the IAT, which has been implemented in thousands of studies over the past decades (26). The IAT relies on the slower behavioral association of incongruent (e.g. outgroup good) vs. congruent (e.g. outgroup bad) pairs of stimuli and its underlying assumption is that reaction time difference, known as the “IAT effect,” indexes a bias towards outgroup members. Yet, the mental processes underpinning the IAT bias are not fully clear (27), despite studies that measured hemodynamic and event-related responses during the IAT paradigm (26). Recently, we introduced a novel approach to study the neural underpinnings of the IAT that focuses on intrinsic neural oscillations and utilizes magnetoencephalography (MEG). We found that the alpha rhythm was continuously activated across the IAT task and implicated a bottom-up component in the occipital cortex that linked with real-life intergroup dialogue styles and attitudes that promote active engagement in peacemaking (28). Prior research has shown that alpha regulates attention and perception (29, 30) as well as emotions (31), indexes the maturation of empathy systems in the brain (32), and reflects intergroup bias in various neuroimaging paradigms (33–36). As such, alpha activity is a potential marker for assessing the effect of interventions on intergroup bias and dialogue in youth reared in the context of intractable intergroup conflicts.

The current study examined, for the first time, the effects of a dialogue-enhancing youth intervention on the neural underpinning of intergroup bias and tested the impact of such neural change on young adults’ attitudes toward peace 7 years later. Notably, interventions targeting intergroup conflicts have not followed participants over lengthy periods and despite the large number of interventions that have been implemented to reduce intergroup bias among races, groups, and nationalities, and the huge social stakes of the issue, there is currently *no evidence* for long-term effects of any intervention beyond several months (37). In a comprehensive review of 418 experiments on interventions for intergroup bias (37), only 1% of the studies evaluated intervention effects 1 month after the intervention and none examined long-lasting effects. Furthermore, even when participants show decreased negative emotions towards outgroup, this is not translated into a constructive action plan (38). While intergroup bias negatively impacts proactivity in support for peace (28), one of the best indices for supporting peace is the willingness to make compromises and the belief that peace is possible (39–41). Thus, in addition to assessing the effects of intervention on youths’ neural intergroup bias, we tested whether the change in neural response has led to a proactivity toward peacemaking and attitudes of peace-support in the long run when participants are young adults and can assume actual civil duties and responsibilities.

**Fig. 1.**
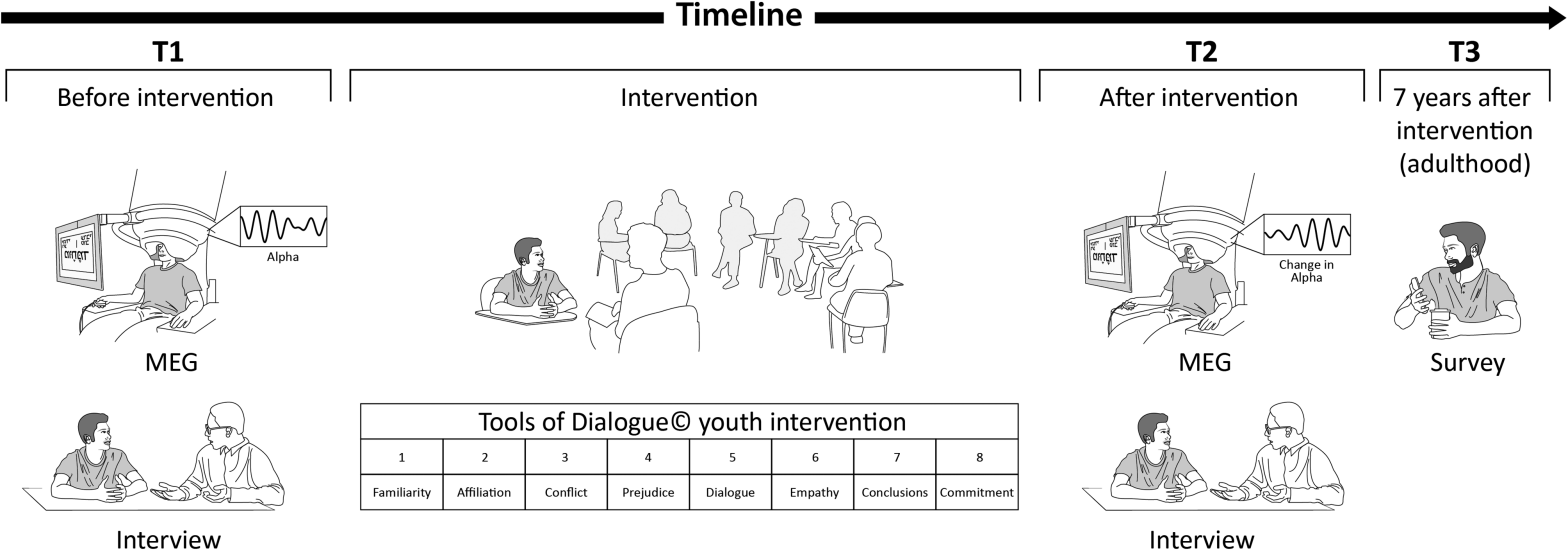
Longitudinal study design. At T1, Jewish–Israeli and Arab–Palestinian adolescents underwent MEG session to assess the neural intergroup bias as represented by the occipital alpha rhythm and one-on-one interviews regarding peace-promoting attitudes. Following, adolescents were randomly assigned to either an 8-week dialogue group intervention or control. As illustrated, in each week of the intervention, group leaders introduced a different topic and provided tools for greater dialogue. In T2, about 5 months after T1, participants came back to do the MEG and interview to evaluate the intervention’s impact. At T3, 7 years after the intervention, a survey assessed young adults’ peace-promoting attitudes.

Framed within the Israeli–Palestinian conflict, the current study applied the Tools of Dialogue© (16), a manualized, validated 8-week dialogue-enhancing intervention based on the neurobiology of affiliation to Israeli and Palestinian youth. The study assessed change in youths’ neural prejudice response from baseline (T1) to post-intervention (T2) and tested the effects of such change on the participants’ peace-promoting attitudes 7 years after the intervention (T3) when they were young adults (Fig. 1). The neural representation of intergroup bias was measured as a quantitative and objective index of intergroup bias using the neural IAT index (28) and complemented by in-depth individual interviews at T1 and T2 and online survey at T3. A cohort of 16 to 18 year-old Israeli and Palestinian youths was recruited and randomized to intervention and control groups. We chose late adolescence to target a period when vulnerability to negative biases toward the outgroup may be at its peak among both Jews and Arabs in Israel; during this period, which is characterized by immature impulse control, individuals are more responsive to incentives and socioemotional pressures, such as training for the military service among Jewish youth or first-time participation in political collective action among Palestinian youth, but without the ability for mental reflection that characterizes adulthood (42). However, late adolescence is also a period when prosocial and moral faculties undergo rapid maturation (43) and this time may provide a potential opportunity for individual transformation. Three hypotheses were tested. First, we expected the Tools of Dialogue© intervention, which included active social coordination and movement synchrony tasks as well as one-on-one and group discussions that focus on affiliation, empathy, prejudice, and dialogical modes of conflict resolution, to attenuate the neural intergroup bias response (44). To test this hypothesis, we repeated two MEG sessions before and several months after the intervention during the IAT task. Second, we expected that the intervention would promote real-life change in attitudes as expressed during in-depth interviews conducted before and after the intervention. Finally, we tested the potential of the intervention to induce long-lasting changes in support for peace and in assuming a proactive peacebuilding approach 7 years after the intervention, thereby charting an adolescence-to-adulthood neural-attitudinal pathway.

## Results

**Fig. 2.**
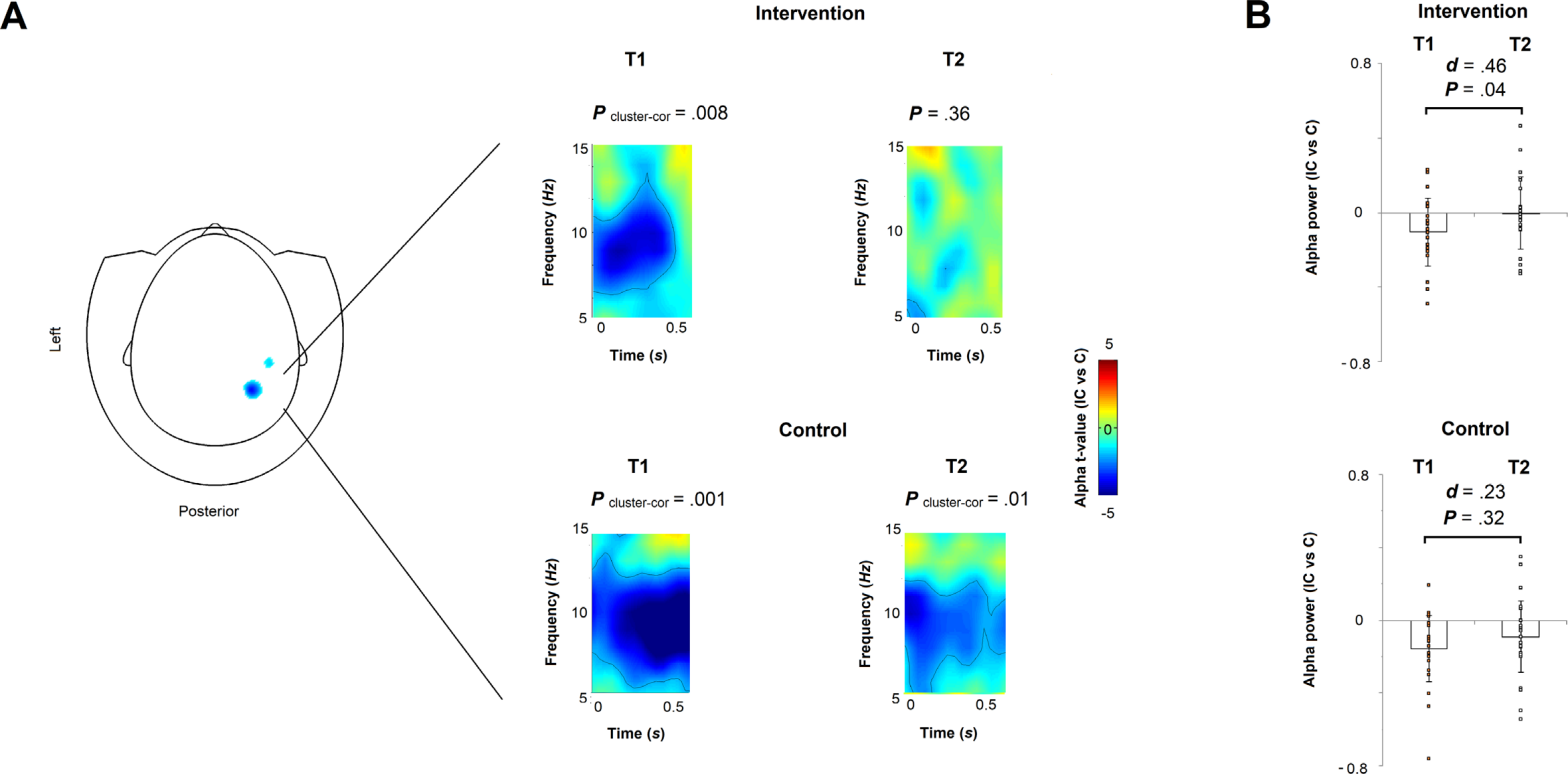
Neural intergroup bias before and after dialogue-enhancing youth intervention. (A) Left panel topographical illustration of the head and the MEG helmet depicts the peak sensors (in blue) involved in the neural intergroup bias effect. On the right, time‐frequency representation maps illustrate induced oscillatory activity (5 to 15 Hz; 0 to 0.55 seconds) filtered from the peak sensor across the two experimental groups at T1 and at T2. (B) Means ± SD of the neural intergroup bias extracted from the peak sensor in the alpha-band in the two experimental groups at T1 and at T2. The difference in the alpha suppression effect was statistically significant (*P* = 0.04) and medium-sized (*d* = 0.46) before and after the intervention, whereas it was insignificant (*P* = 0.23) and small-sized (*d* = 0.23) in the control group.

At T1, we examined IAT data first at the behavioral level using the typical RT measures for IAT trials. As expected, during the incongruent (IC) and congruent (C) conditions, participants responded significantly [*P* = 0.00005, *t*(44) = 4.24] slower in the first (*M* = 1000.35, *SD* = 179.86 ms) compared to the second (*M* = 851.57, *SD* = 151.53 ms) condition, demonstrating the IAT effect at the behavioral level. At the neural level, IAT also had a clear effect, which was revealed by contrasting the IC with the C condition and was expressed as alpha suppression peaking at 100 to 550 ms and at 9 Hz under right posterior MEG sensors (Fig. 2A).

Following the intervention at T2, approximately 5 (*M* = 4.68, *SD* = 2.07) months; no significant difference between two groups [*t*(43) = 0.85, *P* = 0.39] months after T1, we reexamined the behavioral (RT) and neural (alpha) measures to evaluate intervention effects. A mixed-design of repeated-measures between-subject ANOVA was conducted first at the behavioral level and showed no significant main or interaction effects [*F*(1,43) < 1.42, *P* > 0.24], thereby suggesting that the behavioral index (i.e. RT) of implicit prejudice is not significantly affected by the intervention. To address the first hypothesis, that the intervention attenuates the neural intergroup bias response, ANOVA yielded significant main effect for time [*F*(1,43) = 4.37, *P* = 0.04, Eta^2^ = 0.09] but not interaction [*F*(1,43) = 0.21, *P* = 0.65] for the neural IAT effect. Post-hoc analyses showed that a significant alpha suppression pattern was present for youth in the control group at both T1 and T2. However, participants in the intervention showed a significant alpha suppression pattern only at T1, but not at T2 after the intervention (Fig. 2A). A second post-hoc analysis revealed that for individuals in the control group the difference between the two assessments—T1 and T2—in the neural index was not statistically significant [*t*(1,22) = -1.02, *P* = 0.32, Cohen’s *d* = 0.23—small effect-size). However, participants in the intervention group showed a significant difference between T1 and T2 assessments [*t*(1,21) = -2.17, *P* = 0.04, Cohen’s *d* = 0.46—medium effect-size]—stronger alpha suppression was observed in this group at the T1 imaging (*M* = -0.10, *SD* = 0.18), compared to T2 post-intervention imaging (*M* = 0.00, *SD* = 0.20) (Fig. 2B). Importantly, the alpha response at T1 was significantly suppressed [*t*(1,21) = -2.65, *P* = 0.01], whereas after the intervention (i.e. at T2), it was not significantly suppressed, that is, not significantly different than zero [*t*(1,21) = -0.05, *P* = 0.96], suggesting that the neural index was robust before but not after the intervention. Thus, our findings confirm our first hypothesis, and suggest that the intervention attenuated implicit intergroup associations expressed as early perceptual alpha rhythm.

We then proceeded to localizing the neural substrates characterizing the IAT effect (IC vs. C); the alpha effect was localized (*P*_cluster-cor_ < 0.05) in the right lingual cortex, thereby confirming the perceptual function of the neural effect investigated here. To examine whether the intervention impacted the neural index at the lingual cortex level, time series were extracted from this cortical patch, and a permutation test on each time sample was conducted while contrasting the two conditions (IC vs C). Alpha suppression was found in the control group regardless of assessment time T1 or T2 (*P*_cluster-cor_ < 0.05). However, in the intervention group, at T1 alpha was not significantly (*P*_cluster-cor_ = 0.07) suppressed, nor was it at T2 (*P*_uncorrected_ > 0.25). Likewise, testing the intervention group difference between T1 and T2 was not statistically significant (*P*_uncorrected_ = 0.10, Cohen’s *d* = 0.27). Altogether, whereas our findings at the sensor level show a robust effect, the source findings do not point to a statistically significant modulation by the intervention in the right lingual cortex. We therefore continued to further investigate the main intervention effect as revealed here—the early alpha effect.

**Fig. 3.**
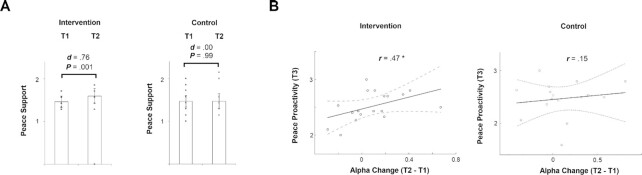
Effects of dialogue-enhancing intervention on peace support in adolescence and adulthood. (A) Means ± SD of Peace Support (obtained from in-depth interview) are represented in the two experimental groups at T1 and T2. The difference in Peace Support was statistically significant (*P* = 0.001) and large-sized (*d* = 0.76) before and after the intervention, whereas it was insignificant (*P* = 0.99) with null effect-size (*d* = 0.00) in the control group. (B) Illustration of Pearson correlation (*r*) between the neural effect (i.e. T2 and T1) and Peace Proactivity at adulthood (i.e. T3) in both intervention and control groups. *P < 0.05.

To address our second hypothesis, that the intervention would enhance attitudes of peace support between T2 and T1, we extended our investigation by examining attitudes via in-depth interviews. To this end, we examined whether the intervention affected *Peace support*, as assessed by individual interviews. A mixed-design of repeated-measures between-subject ANOVA was conducted and revealed significant interaction and main-effect [*F*(1, 39) = 4.80, *P* = 0.03, Eta^2^ = 0.11]. Post-hoc analysis showed that for individuals in the control group, the difference between the two measures (T1 and T2) was not statistically significant [*t*(1,19) = -1 × 10^-9^, *P* = 0.99, Cohen’s *d* = 0.00—no effect-size], whereas in the intervention group that difference was statistically significant [*t*(1,20) = -3.56, *P* = 0.001, Cohen’s *d* = 0.76—large effect-size], so that interviews with adolescents revealed weaker peace support in T1 (*M* = 1.46, *SD* = 0.14) before the intervention, compared to after the intervention at T2 (*M* = 1.60, *SD* = 0.18) (Fig. 3A). Noteworthy, we did not directly contrast the Peace Support measure at T1/T2 with T3 because the first leaned on in-depth interviews whereas the latter relied on a self-reported survey.

Finally, to address our third hypothesis, that is, to test whether the intervention yields long-lasting changes in the participants’ support and active engagement in peace at T3, we examined the *Peace Proactivity* parameter via an online survey. Overall, young adults at T3 revealed a moderate-to-high level of self-reported *Peace Proactivity*. An independent sample *t*-test revealed that there was not a statistically significant difference [*t*(1,33) = 0.43, *P* = 0.66] in this parameter between the intervention (*M* = 2.52, *SD* = 0.26) and the control (*M* = 2.47, *SD* = 0.32) groups. We then proceeded to examine whether the neural intervention effect, the change between T2 and T1 in the neural intergroup bias measure, may serve as index of long-lasting changes at T3. Pearson correlations between the two variables for each group found a statistically significant positive correlation in the intervention (*r* = 0.47, *P* = 0.04) (Fig. 3B) but not in the control group (*r* = 0.15, *P* = 0.56). We also explored, using a mediation model (45), whether there is an indirect effect (via the neural bias measure) of the intervention on *Peace Proactivity*. Results showed that the indirect effect (0.008) was not statistically significant: 95% *CI* [-0.040, 0.097]. Furthermore, although the self-reported measure of *Peace Proactivity* was collected at T3, not at T1 or T2, we considered it important to attempt and control whether the correlation between the neural effect and *Peace Proactivity* may be related to baseline levels of peace support. The closest measure for *Peace Proactivity* at T1 was the interview-based *Peace support*. We therefore repeated the above correlations, while this time controlling for baseline (i.e. T1) levels of peace support. This correlation yielded a significant (*r* = 0.57, *P* = 0.01) correlation in the intervention but not in the control (*r* = 0.23, *P* = 0.39) group. These analyses addressed our third hypothesis and support a unique and surprising finding: whereas no significant group differences persisted 7 years after the intervention, the individual change in the neural prejudice response following the intervention predicted attitudes of compromise and active engagement in peacebuilding 7 years later, plausibly regardless of baseline individual levels of peace support.

## Discussion

Intractable intergroup conflicts are widespread across the globe and lead to intense fear and hatred, atrocious civilian casualties, and perpetually escalating armed conflict (39), underscoring the urgent need for efficient interventions that can mitigate some of their negative outcomes. One of the earliest, most promising interventions was developed by Allport (1954) not long after the end of world-war II and built on face-to-face contact between outgroup members, guided by the assumption that face-to-face contact yields substantial psychological benefits for its participants. Indeed, a meta-analysis of over 500 studies based on the contact approach found that under optimal conditions, that is, equal status between the groups in the situation, common goals, intergroup cooperation, and the support of authorities, law, or custom, direct contact improves intergroup relations (46). Yet, the extensive literature also highlights the limitations of the contact approach and its dependence on optimal conditions, which are rarely found in cases of intractable conflicts (9), and more recent interventions in the context of intractable conflicts target other processes such as intergroup perceptions (41) or emotions (47). Our intervention adopts Allport’s face-to-face contact element; however, based on our extensive research on the role of face-to-face synchrony on shaping the social brain (48, 49) and the *biobehavioral synchrony* conceptual frame, we added several key components. First, we began and ended each meeting with synchronous games involving movement, singing, and ritual, consistent with studies that link movement synchrony with positive affect and connectedness (44). Second, we took time to become familiar with each participant and his/her circle of affiliation; with family, friends, and culture, to highlight the universal role of affiliation in the life of all and its positive impact on the ability to resolve conflict (50). Lastly, we zeroed in on relevant topics related to communication in the context of conflict and provided youth concrete guidelines and opportunities to exchange ideas and feelings, raise issues, and dialogue differences within a containing context and predetermined session topics. For instance, we devoted a session to empathy and how it can be fostered and expressed, another to prejudice and how to recognize it in ourselves and others, and yet another to dialogical versus toxic modes of conflict discussion. Sessions were led by two leaders, Israeli–Jewish and Arab–Palestinian, allowing youth to speak in their native tongue, with the help of translators. We expected such dialogue-based intervention to have a pervasive impact on brain and behavior, induce lasting changes in the neural prejudice response, and promote future support of peacemaking in the context of the Israel–Palestinian conflict and its intense intergroup bias and inflexible attitudes toward peace (51). Our results indicate that the dialogue-enhancing youth intervention attenuated the neural prejudice response, as indexed by the early and sustained alpha rhythms, promoted youths’ orientation toward active engagement in peacebuilding efforts, and predicted attitudes toward peacemaking and compromise in young adulthood 7 years after the intervention.

For decades, social psychologists have studied the impact of intergroup contact on psychological processes (8). Modern intergroup interventions were developed to complement more traditional ones, including diversity training (52), social media (53), cognitive/emotional training (54), and others. Yet, despite this massive effort there is, to date, very little evidence that *any* intervention carries long-lasting effects (37). Paluck and colleagues, in a review of 418 relevant experiments (37), point to a troubling situation: the majority of intergroup interventions exaggerate effects due to small sample sizes and publication bias, show limited effects in the short term, and only 1% of the studies tested effects 1 month or more post-intervention. Even landmark studies show limited effects. For instance, Mousa conducted a sports-based contact intervention in Iraq and despite the effects found on social cohesion in the intervention participants, no consistent impact was found on intergroup attitudes and their translation to other social contexts beyond the soccer team (55). In this context, our approach differs from other intergroup studies in two key features: first, by testing intervention effects on neural processes, and second, by measuring long-term (7 years) outcomes and their association with the neural effects. We show that for the intervention participants, but not for controls, the cortical alpha rhythm underlying the neural prejudice response was significantly attenuated (medium effect-size) and their peace-promoting attitudes were measurably enhanced (large effect-size). The first effect corresponds to cortical activity; hence, the reduction of this rhythm, even if medium-sized, is noteworthy. In parallel, the second effect corresponded to a three-point likert-scale and therefore the large effect-size should be interpreted with caution. Further and importantly, the degree of change in each participant’s occipital alpha rhythm was found to predict attitudes toward peace-making 7 years after the intervention. Such linkage between the degree of neural change following intergroup intervention and long-term outcome is unique and relies on the emerging field of “neuroprediction” (56). It is therefore potentially important at the applied level, for designing neutrally-informed interventions and assessing their impact, as well as at the theoretical level, by charting a brain-attitude correspondence and opening new vistas for utilizing neural markers in research on inter-group relations.

The IAT has been implemented in thousands of studies over the past decades (57). Yet, the IAT paradigm has been under fire and authors debated with regards to the underlying mental processes and intergroup behavior it reflects. The present results, while relying on the IAT, utilize the paradigm in two novel ways. First, it is possible that the neural rhythms underlying the IAT effect may capture mechanisms related to intergroup bias not studied before. Probing the neural, rather than the reaction-time response may yield more consistent associations that can shed new light on the relations between implicit intergroup biases and actual behaviors and attitudes toward outgroup members. Although previous EEG and fMRI studies looked into the mechanisms activated by the IAT (58, 59), our recent study was the first MEG experiment that pinpointed spontaneous frequency-based representations in simultaneous brain regions (28). We found that the cortical alpha rhythm plays a crucial role in sustaining intergroup bias and impacts concrete intergroup experiences. The current findings expand this approach and show that the alpha rhythm during the IAT is significantly reduced after an intergroup dialogue intervention and this effect predicts long-lasting support of peacemaking. Consistent with our findings, Cazzato and colleagues used cathodal transcranial direct current stimulation of the occipital cortex to reduce the IAT effect (60) and similarly showed, using technology rather than dialogue intervention, that altering neural activity in occipital cortex is causally linked with reduction in intergroup bias. The findings from both studies highlight the potential of psychological and technological interventions to alter neural activity and consequently impact intergroup processes. In addition, long-term effects on IAT change has rarely been tested and the few studies that did measure, IAT change was tested hours or days after the interventions (61). Here, the IAT effects were measured several months after the intervention and, consistent with the literature, we found no typical IAT effect. Nonetheless, we report, for the first time, a neural IAT effect following an intergroup intervention and showed that our neural marker of the IAT was significantly reduced in the participants who underwent the intervention. Altogether, both the unique neural marker of the IAT and the repeated assessment several months after the intervention provide the first evidence that dialogue intervention can alter the individual’s neural response in ways that can carry a long-term impact. It is important to note, that our findings would have been more powerful if the long-term impact was assessed at the behavioral level and not only the attitudinal level. Such assessment could have enabled, for example, to test whether the dialogue intervention may lead participants to form more contacts with the other side or engage in more actual dialogue over the years. Future studies should attempt to tackle this point and evaluate concrete behavioral changes induced by the intervention (37).

One aspect that may raise caution in the interpretation of the results is that the intervention impact on the neural IAT index was not detected as a typical interaction effect, but rather as main-effect of prepost in the repeated-measures mixed ANOVA. Typically, one would assume that such plausible interaction would reflect a pre vs post effect in the intervention group that is significantly different than in the control group. However, our findings also showed that there was an average reduction in the IAT effect in the control group, although not statistically significant [*t*(1,22) = -1.02, *P* = 0.32]. Yet, the lack of significant interaction can be mitigated by one recurring observation in IAT studies: repeating the IAT reduces the IAT effect, that is, the IAT effect in the first time is very often larger than that after a second time one conducts the IAT (25). Hence, this suggests that interaction effect in prepost IAT settings would be difficult to observe. Furthermore, one should also take into consideration that in this investigation, the focus should be not only on the ANOVA or *t*-tests (Fig. 2B), but also the presence of the alpha rhythm reflecting implicit bias/prejudice. As seen, this rhythm was present in both groups at the pre stage, but only in the control group at the post stage (Fig. 2A). Our findings show that only participants who underwent the interventions did not show the alpha effect (Fig. 2A), and this is a novel and unique finding. Considering these two important points, the current findings emphasize the observation that only participants who received the intervention exhibited a statistically significant reduction in the neural IAT effect. Nevertheless, it is noteworthy that the sample size in this study is not large and this may have affected the extent of the effects reported here. We have previously shown in a large scale study that during adolescence, the underlying substrates of the alpha rhythm undergo radical changes during the experience of empathy (32). Recruitment of adolescents into our study involved a huge effort; we recruited adolescents from various towns and villages across the country into a single far-away location for multiple sessions, relied on MEG that has tough exclusion criterion, particularly in adolescence (e.g. tooth bracelets), recruited study participants from two rival ethnicities in a climate of conflict, Jewish and Palestinian youth grow up in segregated communities and have never had the opportunity to meet members of the other group, and the need to maintain our participants for a follow-up of 7 years, posed an immense challenge to obtain a larger sample size that would have revealed a stronger effect. The reduced sample size may have affected the examination of the interventional impact at the behavioral level and may explain the lack of significant behavioral effects at T3. Nevertheless, the sample size that we managed to recruit under those challenging circumstances is considered to be a good size in MEG studies that often do not exceed one or two dozens of participants (62–65). Hence, while this further supports the reliability of the current findings, future MEG studies on intergroup interventions are required to further examine the generalizability of the findings reported here.

It has been shown that the alpha rhythm in the brain develop on the basis of prior experiences and maturational stage. Specifically, alpha undergoes a transition from adolescence to adulthood that not only consolidates its suppression, but also shifts its involvement from sensory to affective-cognitive processing. Studies have shown that this transition in the consolidation of alpha enables the maturation of emotion regulation skills by recruiting neural substrates that underlie cognitive functions. Interpolating to our findings, it is possible that adolescence provides a rare and brief window in the course of development wherein interventions can affect neural activity that sustain intergroup bias. In Israel, similar to other war zones of a long and bleeding history, adolescence is a period of vulnerability to the effects of intractable conflict. Jewish–Israeli adolescents prepare for their army service while Arab–Palestinians increase the search for national roots and identify with Intifada activities (66). During this period, adolescents from both ethnicities are particularly susceptible to cognitive biases while shutting down the neural empathic response to the suffering of the outgroup (4). The present study uniquely examines the neural effects of an intervention conducted during adolescence and utilizes the neural change as a predictor to adults’ peacemaking. Our findings, therefore, highlight the utility of this brief window as a sensitive period in development that can provide opportunities for one-on-one encounters with outgroup members, which may modulate the formation of rigid neural representations of outgroup members that may shape future action. Focusing on interventions that combines synchrony with dialogue, targets specific topics in social collaboration, and discusses techniques for prejudice-reduction and the management of group conflict may present a promising novel approach to conflict resolution. Our results chart a hopeful trajectory and open the possibility to attenuate fear and hatred among youth reared within deep-rooted conflicts and describe a potential venue toward greater compromise, dialogue, and resilience.

## Methods

### Subjects

Forty-seven Jewish–Israeli and Arab–Palestinian adolescents were recruited via social media, advertisement in schools and in adolescents’ organizations for a MEG intervention study while controlling for gender and nationality, and were screened for no serious medical, neurological, or psychiatric conditions and for MEG metal-compatibility (body metal-free, including tooth bracelets, implants, piercing, etc). They were all residents in the center of Israel (within a 50 km distance from Tel-Aviv) and citizen of Israel. Arab–Palestinians are a minority group in Israel and typically live in different neighborhoods or towns than the Jewish–Israeli majority, and in the majority of cases, adolescents from the two groups have few opportunities for interpersonal encounters. Furthermore, Arab–Palestinians share a Palestinian collective national identity, which is perceived as a threat by many Jewish–Israelis (67). Following MEG data-acquisition at T1 and at T2 (Fig. 1), two of the control participants were excluded from further analysis due to failure to complete the MEG paradigm. This resulted in a cohort of 45 participants, 59% Israeli–Jewish and 52% Arabs–Palestinians, as well as 45.5% and 52% males in the intervention (*N* = 22) and control (*N* = 23) groups, respectively, and ranging in age from 15 to 18 years (*M* ± *SD*, 16.50 ± 0.82).

The study at T1 and T2 received approval from the Bar-Ilan University ethics committee and participants’ parents gave written informed consent before the experiment in line with Bar-Ilan University’s Institutional Review Board. Youth were informed that they can leave at any point during each session or drop out of the study. The follow-up survey at T3 received approval from the Reichman University School of Psychology ethics committee. Subjects in the three phases received monetary compensation for their participation.

### Intervention and study design

Following a randomized controlled trial design, subjects who were randomly assigned to undergo the Tools of Dialogue© intervention (15) participated in weekly 2.5-hour sessions for eight consecutive weeks, led by two experienced professionals, a Jewish–Israeli and an Arab–Palestinian. Because for some individuals there was language barrier to dialogue with each other, translators have been assisting to bridge the language barrier. The aim of the intervention was to foster empathy and understanding between the two ethnic groups, and as such, group leaders encouraged bringing up tension-eliciting topics while providing empathic feedback. Sessions included activities or games that addressed conflictual topics and prejudice via sharing of personal and familial suffering related to the conflict via dyadic and group dialogues. Other activities included role play, group singing, mutual videos and dialoging venues for compromise between the two ethnic groups. The last activity in the intervention was a metaphorical “gift giving” to the group. For pre and post-intervention assessment, identical visits were conducted at baseline (T1) and after intervention or after approximately 5 months for controls (T2), without statistically significant difference (*P* = 0.39) in time-lag (*M* ± *SD*, 4.68 ± 2.07) between T1 and T2 across groups. To assure blind assessment, intervention leaders or assistants did not participate in data collection, coding, or analysis and all information was kept masked until the end of trial and data analysis stage. Each assessment included MEG, in-depth interview, self-reports, one-on-one interactions, and hormonal collection. The hormonal and relational findings are reported elsewhere (14, 15) and were therefore not included in the present study which focused on neural findings from a subsample that was compatible with the MEG neuroimaging requirements. At T3, about 7 years (*M* ± *SD*, 6.8 ± 0.7) after the first visit (T1), we contacted the participants (22.22% attrition rate, while maintaining a 51%/49% balance between intervention/control) to assess peace-supporting attitudes and ideology.

### Attitudes

#### Peace support

At T1 and T2, we conducted an in-depth structured interview with each participant on the topic of the Israeli–Palestinian conflict (28). A qualitative–quantitative transformation was then conducted by the interviewers by rating participants’ attitudes toward each item on a 3-point scale ranging from 1 (strong opposition) through 2 (weak endorsement) to 3 (full endorsement). In the context of the current study, we computed a measure of *Peace support* by averaging seven items that describe the degree to which participants endorsed intergroup: dialog, compromise, acceptance, empathy, and hope for peace. So for example, if a participant, during the interview, expressed high support for dialogue and low support for compromise in the context of the conflict, then the scores 3 and 1 would be given for those two items. This was conducted for all seven items, and then each participant received a single average score (ranging from 1 to 3) reflecting peace support. Although many previous studies evaluated peace support using an excellent questionnaire [e.g. (68, 69)], the approach that was favored here relied on an in-depth interview and then a qualitative–quantitative transformation to rate individual peace support as captured by the rich subjective expressions during the interview. In addition to relying on rich subjective expressions, this approach potentially may reduce the chances for a desirability or a presentability bias during the interviews.

#### Peace proactivity

At T3, we conducted an online survey to evaluate similar parameters (dialog, compromise, acceptance, and hope for peace) as those in the *Peace support* construct, but in addition, we were motivated by our recent report that emphasized being proactive in those processes to achieve peace (28). Hence, the online survey contained eight items (Table 1) that were formulated to tap into the process of being proactive to support peace by rating the items on a 3-point scale ranging from 1 (strong opposition) to 3 (full endorsement).

**Table 1.** The *Peace proactivity* construct.

**What is needed to reach conflict resolution and peace:**
1. Dialogue between the two sides.
2. Dialogue between the two hawkish camps, and potentially resorting to referendum.
3. Stop fighting or the use of force (n.b., reverse-coded).
4. Our side must take responsibility and resolve the conflict via peace.
5. The Jewish side must be active by territorial concessions, education, and awareness.
6. The Arab side must be active by stopping violence, concessions, education, and leadership.
**I believe:**
7. There is solution to the conflict (n.b., reverse-coded).
8. Eventually Jews and Arabs will attain peace.

This score was computed by averaging the eight items in the table.

### MEG recordings and experimental procedures

In two separate sessions (T1 and T2), participants completed the IAT (70) while we recorded ongoing brain activity (sampling rate, 1017 Hz, online 1 to 400 Hz band-pass filter) using a whole-head 248-channel magnetometer array (4-D Neuroimaging, Magnes® 3600 WH) inside a magnetically shielded room. T1 data from 42 of the participants were analyzed to evaluate the neural underpinning of the IAT regardless of any manipulation (i.e. intervention) (28). The E-prime software (Psychology Software Tools Inc.) was used to deliver the IAT stimuli in Hebrew or in Arabic, as a function of participants’ mother tongue, on a screen inside the MEG room while participants lay in supine position. In total, 320 IAT trials were applied to evaluate the IAT bias. More details on the technical procedures of the IAT inside the MEG can be found in the “Methods” section of our previous publication (28).

### Data preprocessing and MEG sensor-level analysis

Data cleaning and preprocessing was performed as detailed in the “Methods” section of previous studies. We then segmented the data into 2500 ms (baseline period: 500 ms) epochs corresponding to the IAT event trials in alignment to stimulus onset, and retained for analysis only trials with response time longer than 300 and shorter than 3000 ms, following IAT analysis recommendations (25). Epochs were filtered at 1 to 200 Hz range with 10 seconds padding and then resampled them to 400 Hz. We first computed the typical D’ index, a behavioral index of the IAT effect, taking into account the difference in response time between the IC and C conditions and the number of errors made, according to IAT analysis guidelines (25). We then applied tapers to each epoch to compute Time-Frequency Representations (TFRs) of power and to calculate the fast Fourier transform (FFT) for short sliding time windows, and then averaged the power estimates across tapers. A Hanning taper, applied to each epoch of the sensor data, yielded the FFT for short sliding time windows of 0.5 seconds in the alpha band (8 to 12 Hz). We obtained induced activity by subtracting evoked-components’ power from oscillatory power. Analyses were performed using MATLAB (MathWorks, Natick, MA, USA) and the FieldTrip software toolbox. Correlations between neural and behavioral data applied Pearson’s *r*, and statistical significance of the power values was assessed using a nonparametric permutation procedure (71).

## Data Availability

Data and code that support the findings of this study are available under ethics restrictions and on reasonable request from the corresponding authors (yoniilevy@gmail.com). The data are not publicly available due to them containing personal information that could compromise research participants’ consent.
