# Correction to: Dialogue intervention for youth amidst intractable conflict attenuates neural prejudice response and promotes adults’ peacemaking

**DOI:** 10.1093/pnasnexus/pgad474

**Published:** 2024-01-30

This is a correction to: Jonathan Levy, Moran Influs, Shafiq Masalha, Abraham Goldstein, Ruth Feldman, Dialogue intervention for youth amidst intractable conflict attenuates neural prejudice response and promotes adults’ peacemaking, *PNAS Nexus*, Volume 1, Issue 5, November 2022, pgac236, https://doi.org/10.1093/pnasnexus/pgac236

During a retroactive audit conducted by *PNAS Nexus*, it was discovered that this paper was missing a statement acknowledging compliance with the *PNAS Nexus* Human and Animal Participants and Clinical Trials policy:

The participants of the follow-up survey at T3 gave written informed consent before the experiment.

This error has been corrected in the original article.

